# Supplementary material for: ICRA: A study of highly accurate course recommendation models incorporating false review filtering and ERNIE 3.0
Source: PLoS One. 2024 Dec 11;19(12):e0313928. doi: 10.1371/journal.pone.0313928 (PMC11633988; doi:10.1371/journal.pone.0313928)
Supplement: Attachment — Submitted filename: Response to Reviewers.docx [file pone.0313928.s001.docx]

**Original Manuscript ID:** PONE-D-24-27196

**Original Article Title:** “ICRA: A Study of Highly Accurate Course Recommendation Models Incorporating False Review Filtering and ERNIE 3.0”

Dear Editors and Reviewers,

Thank you for giving us this valuable opportunity to submit a revised version of our manuscript titled "[ICRA: A Study of Highly Accurate Course Recommendation Models Incorporating False Review Filtering and ERNIE 3.0]" (Manuscript ID: PONE-D-24-27196). We sincerely appreciate the detailed and constructive comments provided by the reviewers. We have carefully revised the manuscript by incorporating all the reviewers' suggestions. Compared to the original manuscript, we have added some figures and explanations, and have thoroughly checked and corrected spelling and grammatical errors in the manuscript. We hope that the revisions in the manuscript, as well as our point-by-point responses to the reviewers' comments, will make our manuscript suitable for publication in PLOS ONE.

Attached to this letter is our point-by-point response to the reviewers' comments. For ease of discussion, we first restate your comments in blue, followed by our responses. Additionally, the changes in the manuscript are highlighted in red.

We look forward to hearing from you at your earliest convenience.

Yours sincerely,

Bing Li et al.

**Reponses to Editor**

Thank you for your valuable feedback and suggestions on our submitted manuscript. Based on your comments, we have made the following revisions to the manuscript:

1. **Formatting Requirements：**We noted that the current manuscript format does not meet the accepted file types for PLOS ONE. In response, we have reformatted the manuscript as a .tex file (with an accompanying .pdf) and have resubmitted it. Please review the updated files at your convenience.
2. **Code Sharing Guidelines：**We have checked and ensured that all codes related to the research results are publicly accessible and comply with PLOS ONE's code sharing guidelines. To ensure the reproducibility and reusability of the research, we are providing the access link to the code: <https://github.com/hyq9/ICRA>
3. **Dataset Information and Statement：**Thank you for your valuable feedback on my manuscript. In response to your suggestions, we have added detailed information about the dataset in the Methods section of the manuscript(Data collection, page 6), along with a statement confirming that our data collection and analysis processes strictly adhere to the terms and conditions of the data source to ensure legal and regulatory compliance.
4. **Correction of Funding Information：**We have carefully reviewed the "Funding Information" and "Financial Disclosure" sections to ensure the correct grant numbers are listed. All funding-related content has been removed from the acknowledgments section of the manuscript.
5. **Funder Role Statement：**The funder role statement has been updated. We have included the following new statement in the cover letter: "The funders had no role in study design, data collection and analysis, decision to publish, or preparation of the manuscript." This statement has also been reflected in the revised manuscript.
6. **Removal of Funding Information from the Manuscript：**Funding-related content has been removed from the acknowledgments section of the manuscript. Complete funding information is now provided in the "Funding Statement" section of the online submission form, which has been updated according to your request.
7. **Data Availability Statement**：Thank you for your recommendation regarding the data repository. To comply with PLOS’s data storage standards, we have uploaded the research dataset to the recommended public repository Figshare. The dataset’s access link and DOI are as follows: <https://doi.org/10.6084/m9.figshare.27301371.v1>
8. **Reference List**：We have thoroughly reviewed and updated the reference list to ensure its completeness and accuracy. For retracted articles, we have either provided the reason for citation in the main text or replaced them with current relevant literature. Additionally, if a retracted article was cited, we have clearly marked its retracted status in the reference list.

**Reponses to Reviewer #1**

**Comment 1:**

Please provide the full form of the acronym MOOC when it is first mentioned in the text.

**Response 1:**

Thank you very much for your reminder. We have already provided the full name of MOOC, "Massive Open Online Courses," when it is first mentioned to ensure that readers can clearly understand the term.

**Revised 1 (Introduction, Page 1):**

*Concurrently, the proliferation of MOOC(Massive Open Online Courses) platforms like Coursera, edX, and Udacity has presented learners with numerous course options, posing both opportunities and challenges.*

**Comment 2:**

It is recommended to place Table 3 after its corresponding explanation, similarly for Figure 2.

**Response 2:**

Thank you for your valuable suggestions. We have adjusted the placement of Table 4 (formerly Table 3) and Figure 2 based on your comments, positioning them after the corresponding explanations. In addition, we have refined the surrounding text to ensure coherence and logical flow, making it easier for readers to follow the discussion. Please refer to Revised 2 for the adjustments made to Figure 2 and Revised 3 for the modifications to Table 4.

**Revised 2 (Introduction, Page 3):**

*Fig 2 compares traditional recommender systems with the ICRA recommendation model. Traditional recommender systems primarily rely on user reviews and ratings, but inconsistencies between reviews and ratings often affect system reliability. To address this, the ICRA model integrates false review filtering with course overview analysis, improving recommendation accuracy and reliability.*

**Comment 3:**

In Table 3, please clarify the meaning of the "Improved" column.

**Response 3:**

Thank you for your thorough review of our paper. In response to the question regarding the "Improved" column in Table 4 (formerly Table 3), we have made the following adjustments in the revised manuscript: The original table used the "Improved" column to display the performance improvements of each model in terms of RMSE and MAE on raw and real data, but the numerical presentation may not have been sufficiently intuitive. In the revision, we replaced the "Improved" column with a "Difference" column, which directly shows the differences between the raw data and real data for both RMSE and MAE. Additionally, we have included a heatmap that visually represents the performance changes of different models using color gradients. These adjustments help to more clearly illustrate the performance changes of each model before and after filtering fake reviews, making it easier for readers to intuitively understand the extent of model improvements.

**Revised 3 (Comparison experiment，Page 13):**

*Table 4 compares the RMSE and MAE of each model on the original and real datasets, demonstrating performance improvements. The "Difference" column represents the discrepancy between the original and real datasets, reflecting the performance changes after filtering out false reviews. The last row, "Improved" shows the percentage improvement of the IORA model compared to the historically best methods. On the original dataset, IORA's RMSE improved by 4.31% over ANT, while MAE improved by 2.54%. On the real dataset, RMSE improved by 6.73% over ANT and MAE improved by 2.08% over BERT. The heatmap in Fig 6 provides a visual representation of model performance, where darker colors indicate better performance. Most models showed improvements on the real dataset, with the IORA model showing the most significant RMSE and MAE improvements, as highlighted by the dark blue regions. These results demonstrate that the IORA model achieved significant progress in predictive accuracy, particularly in addressing false reviews.*

*Compared to ICRA, the improvements in other models are relatively modest. DeepCoNN and MPCN show notable performance gains on real data after filtering out false comments, whereas PREEI exhibits minimal improvement, suggesting limitations in adaptability. The strong performance of the ANT model underscores the effectiveness of its attention mechanism in managing complex recommendation tasks.*

*The ICRA model’s improvements in RMSE and MAE surpass those of most other models, demonstrating its robustness and accuracy in course recommendation tasks. This indicates that ICRA effectively captures deep user-course interactions, delivering precise recommendations even in challenging, noisy datasets.*

**Comment 4:**
The legend in Figure 6 uses the terms "Actual" and "Original Data," which differ from the term "Real data" used throughout the paper. Please standardize the terminology for consistency.

**Response 4:**

Thank you for pointing out the inconsistency. We have made adjustments in the revised manuscript to the legend of Figure 7 (formerly Figure 6) and the corresponding content in the text, consistently using the term "Real data" throughout to ensure uniformity and avoid reader confusion.

**Revised 4 (Ablation experiment, Page 15):**

**Reponses to Reviewer #2**

**Major comments 1:**

It’s not clear if the authors evaluate their method in which of the following settings:

a. Given a new user, find the most appropriate course recommendation? - If this is the case, the recommendation should be conditioned on some kind of user query/need. In addition. The model should be evaluated using standard recommendation model metrics such as precision@k, recall@K, Hit rate, Mean reciprocal rank (MRR) etc.
Vs
b. Given a course profile and its reviews , determine what is the rating the course should get? - if this is the objective, the proposed model should be presented as a course rating model and not a course recommendation model. (which I believe is the case)

**Response 1:**

Thank you very much for your thorough review of our manuscript, especially for the specific suggestions regarding the evaluation settings. Your feedback has been extremely valuable in improving the content of our paper. In response to the model positioning issue you raised, we have made the following clarifications and revisions:

Firstly, the primary goal of our model is to predict the rating a course should receive based on course materials and user reviews. From this perspective, our model aligns more with the definition of a course rating prediction model rather than a traditional Top-K recommendation system. By performing deep semantic analysis of course reviews, we can accurately predict user ratings for the courses. Therefore, in the model evaluation, we used RMSE and MAE as the main metrics to measure the accuracy of rating predictions, rather than the evaluation metrics commonly used in Top-K recommendation systems (such as precision@k, recall@k, etc.).

Additionally, recommendation systems can be categorized based on different task scenarios, such as rating prediction, Top-K recommendation, click-through rate prediction, context-aware recommendation, and group recommendation. Each task corresponds to different evaluation metrics: rating prediction typically uses RMSE and MAE, Top-K recommendation uses Precision@K, Recall@K, NDCG, and click-through rate prediction commonly uses AUC and CTR. The nature of the task determines the choice of evaluation metrics, which helps comprehensively assess the performance of the recommendation system. In rating prediction tasks, RMSE and MAE effectively reflect the error between predicted and actual ratings. Lower RMSE and MAE values indicate smaller prediction errors, thus indicating better model performance.

However, as you mentioned, rating predictions can also be further used to recommend suitable courses to users. In the ICRA model, rating prediction is one of the key steps to improving recommendation accuracy, rather than the final goal. Therefore, the model not only predicts ratings but can also recommend the best courses to users by ranking or filtering based on the predicted ratings. By predicting ratings for different courses, the model helps users make more informed decisions and thus has a certain recommendation system function.

Although we have not yet used standard recommendation system evaluation metrics, we plan to further expand the model’s functionality in the future to generate more personalized course recommendations by incorporating users' historical behaviors and preferences. In the revised manuscript, we have adjusted the model architecture and conclusion sections to more clearly demonstrate the model's dual functionality: not only as a rating model but also as a tool to provide personalized course recommendations to users.

Once again, thank you for your valuable feedback. Your insights have been crucial in helping us improve the content of the paper. We look forward to presenting the full functionality of the model and addressing the issues you raised in the revised manuscript.

**Revised 1:**

**(Model architecture, Page 9)**

*Rating prediction is one of the core problems in personalized recommendation systems, aiming to score the interactions between users and courses. The ICRA model predicts user ratings for unseen courses, providing a basis for personalized ranking in the recommendation system. The model predicts scores for all candidate courses and ranks them based on the predicted scores, prioritizing those with higher ratings to achieve personalized recommendations. The ICRA model leverages data collected from the Chinese University MOOC platform, combined with a self-built sentiment lexicon and the pre-trained ERNIE 3.0 model, to conduct in-depth semantic analysis. This model not only accurately identifies and filters fake reviews, ensuring high-quality datasets, but also predicts user ratings for new courses based on their historical rating records. To achieve this, the ICRA model consists of an embedding layer, a feature processing layer, a regularization layer, and an output layer, all of which work closely together to improve rating prediction accuracy.*

*Figure 5 illustrates the overall architecture of the ICRA model, where* $C_{u}$ *represents the user comment document,* $C_{i}$ *represents the course overview document,* $E_{u}$ *and* $E_{i}$ *are the embedding matrices for user and course comment documents, respectively, and* $y_{i}$ *and* $\hat{y_{i}}$ *denote the actual and predicted ratings of users for courses.*

**(Conclusion, Page 16)**

*Despite the strong performance of the ICRA model on MOOC platforms, it has some limitations. It was developed based on data from the Chinese University MOOC platform, and its generalization to other online learning platforms remains unverified. Additionally, the false review detection module relies on existing sentiment lexicons and the ERNIE 3.0 model, which may have limited accuracy in identifying inauthentic reviews. Furthermore, the complexity of the model and its high computational demands may limit its application in resource-constrained environments.*

**Major comments 2:**

The presence and filtering of ‘false reviews’: Authors do not present any statistics/ citations to support their argument about wide spread prevalence of fake reviews in online courses. More importantly, there is no evaluation provided to determine if the ‘false comments recognition’ module does in fact only remove fake reviews or just filters random reviews. While an argument can be made that the review filtering does improve performance, if it is in fact removing false reviews seems a claim that should be substantiated with better analysis.

**Response 2:**

Thank you for your detailed feedback and valuable suggestions on our paper. We recognize the lack of sufficient statistical data and citations to support our argument regarding the prevalence of fake reviews. Furthermore, the analysis of the effectiveness of the fake review detection module is indeed insufficient, as it fails to demonstrate whether the module only removed fake reviews rather than random reviews. In response to your comments, we plan to make the following improvements in the revised manuscript:

1. **Citing Relevant Research and Statistical Data:** In the revised manuscript, we will cite relevant research and reports, providing statistical data to support the prevalence of fake reviews on online education platforms (such as MOOC platforms). The data will come from published surveys and market analysis reports. For example, studies have shown that many online review platforms have a significant proportion of fake reviews. Approximately 16% of reviews on the popular U.S. restaurant review site Yelp are fake, and this figure rises to 42% on Amazon. According to the "China Internet User Rights Protection Survey Report" published by the China Internet Association, 72.7% of consumers have encountered fake reviews. The "2021 Fake Online Reviews Report" jointly released by the cybersecurity company CHEQ and Professor Roberto Cavazos from the University of Baltimore stated that fake reviews were expected to directly impact $152 billion in consumer spending in 2021. This data will provide a stronger theoretical basis for the necessity of the fake review detection module.
2. **Fake Reviews in Online Education:** We found that online education, as part of the online goods category, also faces the threat of fake reviews. Fake reviews in online education are more subtle, often exaggerating the quality of the courses, the qualifications of the teachers, and the learning outcomes. For example, while analyzing the MOOC dataset, we observed several anomalies, as shown in the table below: Some positive reviews were accompanied by low ratings, negative reviews had high ratings, and some reviews consisted only of punctuation marks or meaningless gibberish. Moreover, we noticed that certain users repeatedly used similar phrases to review the same course. These anomalies all point to the existence of fake reviews. The purpose of fake reviews is to attract more students to enroll or to harm competitors' courses through malicious reviews. In addition, there are cases where teachers or institutions manipulate students to post favorable reviews or boost course enrollment. These behaviors, by manipulating ratings and review content, not only mislead other users but also affect the accuracy of the recommendation system, ultimately harming the user’s learning experience.

3. **Improvement of the Fake Review Identification Module:** In response to your concerns, we will provide a more detailed description of the working principle of the fake review identification module to ensure that it accurately removes fake reviews rather than randomly deleting them. This study identifies fake reviews through three aspects: (1) For cases with inconsistent ratings, we perform sentiment analysis to compare course ratings with review content, generating a sentiment-based rating. If this rating does not match the actual rating, it is marked as a fake review. (2) We eliminate meaningless content and overly short reviews based on the length of the comments and the use of letters and symbols. (3) By analyzing the similarity of textual content, we identify and exclude duplicate or highly similar reviews. These methods combined ensure that fake reviews can be effectively identified and filtered, minimizing the risk of mistakenly deleting normal comments. Ultimately, we identified and filtered 62,888 fake reviews through this module, accounting for approximately 11.37% of the total data. This not only improves the accuracy of the course recommendation system but also provides users with more authentic and reliable learning information.

We sincerely thank you for your valuable feedback on our work; your input is crucial for us to enhance the content of our paper. We will further demonstrate the effectiveness of the model in the revised manuscript and address the relevant issues you raised.

**Revised 2:**

**(A study of fake reviews, Page 5)**

*Research indicates that many online product review platforms are plagued by fake reviews. Approximately 16% of reviews on Yelp and 42% on Amazon are estimated to be fake[25]. According to the "China Internet Users' Rights Protection Report", 72.7% of consumers have encountered fake reviews[26]. Fake reviews not only mislead consumer decisions and disrupt fair market competition, but also undermine platform credibility and lead to significant economic losses. The "2021 Fake Online Review Report," jointly released by CHEQ and University of Baltimore Professor Roberto Cavazos[27], estimated that fake reviews affected $152 billion in consumer spending in 2021. Academic research primarily focuses on the motivations, identification methods, and impacts of fake reviews. Online Deceptive Reviews (ODRs) are harmful to the e-commerce ecosystem due to factors such as emotional venting, incentive-driven reviews, perfunctory comments, and "water armies" manipulating merchant reputations[28]. To tackle fake reviews, researchers have proposed innovative approaches, such as n-gram and sentiment scoring-based intelligent systems[29], sentiment analysis integrating habit bias and XGBoost algorithms[30], and methods like the ERNIE pre-trained model and convolutional neural networks[31].*

*As a part of online products, online education is also threatened by fake reviews. Recommender systems rely on user ratings and other metrics, and fake reviews can significantly undermine the accuracy and reliability of recommendation results. Competitors may post fake reviews to discredit rival courses or artificially inflate their own ratings, misleading learners in their decision-making. Institutions or individuals might also employ "water armies" to post fake positive reviews to attract more learners. These actions not only harm learners' interests but also weaken the credibility of the recommendation system.Applying false review identification technology to course recommendation systems is essential for ensuring accuracy, protecting learners' interests, and fostering a fair and reliable online education environment.*

**(Fake comment identification, Page 8)**

*Through the analysis of the dataset, we identified several anomalies, as shown in the table 3: some positive reviews were accompanied by low ratings, while, conversely, some negative reviews were given high ratings. There were also cases where reviews consisted solely of punctuation marks or meaningless characters. Additionally, we observed that certain users repeatedly used similar wording to review the same course. To address these issues, the fake review detection module consists of three main components, employing a multidimensional approach to effectively identify fake reviews:*

- ***Inconsistency between ratings and review content:*** *First, the module uses sentiment analysis to compare the course rating with the review content, generating a sentiment-based rating. If there is a significant discrepancy between this rating and the actual rating, the review is flagged as a potential fake.*
- ***Meaningless or excessively short reviews:*** *Next, the module filters reviews based on their length and the use of symbols. Reviews that are too short[34], contain abnormal symbols, or include garbled text are considered meaningless or superficial and are thus removed.*
- ***Highly templated or duplicate reviews:*** *Lastly, the module applies text similarity analysis to identify highly similar or duplicate reviews. The ERNIE 3.0 model is used to perform deep semantic encoding of the review text, which is then tokenized using the BERT tokenizer for further processing. The Annoy library is employed to build an approximate nearest neighbor index, enabling efficient similarity comparisons to detect duplicate reviews.*

*By combining review length, sentiment analysis results, and similarity analysis, the module can accurately detect and filter fake reviews. All evaluation results are recorded and stored for further screening and analysis. The module structure is shown in Fig 4.*

**Major comments 3:**

Evaluation: The authors present their evaluation results and list “Improvement percentage” in the last line of Table 3 .The improvement percentage however is calculated by calculating “Average scores from all other benchmarks”. This is a gross misrepresentation of improvement data. Improvement is always calculated from the next best performing method and not an average of the chose benchmarks. Authors are strongly advised to update the manuscript to reflect the improvement from the best performing baseline method, which seem to be in the range of 2-5%.

**Response 3:**

We sincerely appreciate your thorough review and valuable suggestions regarding our work. We understand your concern about the calculation method for the "Improvement Percentage" in Table 4 (originally Table 3). Based on your feedback, we recognize that the current calculation method is flawed, as basing the improvement percentage on the average of all benchmarks is indeed not accurate. As you pointed out, the improvement should be calculated based on the best-performing comparison method.

We have revised the manuscript, recalculated, and updated the improvement percentages to ensure they are based on the performance of the optimal baseline method. Additionally, we replaced the "Improvement" column with a "Difference" column, which directly displays the differences between the model's performance on the original data and the data after filtering out fake reviews, listed by RMSE and MAE. To further enhance the clarity of the results, we also added a heatmap that visually represents the performance variations of different models, helping readers better understand the improvements before and after filtering out fake reviews.

These adjustments make our results more accurate and intuitive. Once again, thank you for your valuable suggestions regarding our research!

**Revised 3 (Comparison experiment, Page 13):**

*Table 4 compares the RMSE and MAE of each model on the original and real datasets, demonstrating performance improvements. The "Difference" column represents the discrepancy between the original and real datasets, reflecting the performance changes after filtering out false reviews. The last row, "Improved" shows the percentage improvement of the IORA model compared to the historically best methods. On the original dataset, IORA's RMSE improved by 4.31% over ANT, while MAE improved by 2.54%. On the real dataset, RMSE improved by 6.73% over ANT and MAE improved by 2.08% over BERT. The heatmap in Fig 6 provides a visual representation of model performance, where darker colors indicate better performance. Most models showed improvements on the real dataset, with the IORA model showing the most significant RMSE and MAE improvements, as highlighted by the dark blue regions. These results demonstrate that the IORA model achieved significant progress in predictive accuracy, particularly in addressing false reviews.*

*Compared to ICRA, the improvements in other models are relatively modest. DeepCoNN and MPCN show notable performance gains on real data after filtering out false comments, whereas PREEI exhibits minimal improvement, suggesting limitations in adaptability. The strong performance of the ANT model underscores the effectiveness of its attention mechanism in managing complex recommendation tasks.*

*The ICRA model’s improvements in RMSE and MAE surpass those of most other models, demonstrating its robustness and accuracy in course recommendation tasks. This indicates that ICRA effectively captures deep user-course interactions, delivering precise recommendations even in challenging, noisy datasets.*

**Major comments 4:**

The paper lacks following details in methods and experimental setup, which makes it hard to follow the results and conclusion:
a. The experimental setup does not provide any details on what is y_true and what is y_pred in context of their evaluation.
b. The ablation designs are not explained at all. They are just listed as “Ours-biLSTM” and “Ours- Attention” with no explanation of what layers of the model were dropped or changed to design ablation study.
c. Figure 5 Model architecture: Its not clear what is in the user embedding in Figure 5 and how is it constructed using filtered reviews. Did the authors mean “review/comment embedding” instead of “user embedding”

**Response 4:**

Thank you for your valuable comments on our paper. In response to your concern regarding the lack of details in the methods and experimental setup, we have made the following additions and improvements:

1. **Regarding the definitions of** $\boldsymbol{y}_{\boldsymbol{true}}$ **and** $\boldsymbol{y}_{\boldsymbol{pred}}$ **in the experimental setup:** In the Experimental Setup section, the definitions of $y_{i}$ and $\hat{y_{i}}$ that we provided are actually the same as those of $y_{true}$ and $y_{pred}$. Specifically, $y_{true}$ corresponds to the actual ratings given by users $y_{i}$, while $y_{pred}$ corresponds to the predicted ratings by the model $\hat{y_{i}}$. We will clarify this point in the revised manuscript to avoid confusion and ensure the definitions are clear and understandable.
2. **Explanation of Ablation Design:** Thank you for pointing out the shortcomings in the explanation of the ablation experiment design. We indeed only listed "Ours-biLSTM" and "Ours-Attention" without adequately explaining the specific model modifications. In the revised version, we will provide a detailed description of the ablation experiment design, indicating that in "Ours-biLSTM," we removed the BiLSTM layer while retaining the multi-head attention mechanism; in "Ours-Attention," we removed the multi-head attention mechanism and kept only the BiLSTM layer. This design aims to explore the impact of different modules on model performance. We will include detailed explanations of the ablation design in the manuscript.
3. **Issue with Model Structure in Figure 5:** Regarding the "user embedding" in Figure 5, our expression here is inaccurate. In fact, it should refer to "review/comment embedding" instead of "user embedding." The review embedding is generated based on the filtered review content. We will correct this terminology in the manuscript and provide a detailed explanation of how the embedding is generated, including how to construct the model's input features from the filtered review content.

Once again, thank you for your detailed feedback; these modifications will help us further enhance the clarity and scientific rigor of the paper.

**Revised 4 (Comparison experiment, Page 13):**

**(A，Experimental setup, Page 12):**

*The experimental data were divided into original and real datasets, with the original dataset containing 552,901 comments and the real dataset consisting of 490013 comments after filtering out fake reviews. These datasets were primarily used for rating prediction tasks. To evaluate the model’s performance, RMSE (Root Mean Squared Error) and MAE (Mean Absolute Error) were used as the main evaluation metrics. Lower RMSE and MAE values indicate higher prediction accuracy, providing a comprehensive assessment of the algorithm’s performance. The formulas are defined as follows:*

*Here, N represents the number of samples,* $y_{i}$ *denotes the actual rating for sample i, and* $\hat{y_{i}}$ *signifies the predicted rating for sample i. These evaluation metrics provide a systematic and comprehensive assessment of the recommendation algorithm’s accuracy and performance.*

**(B，Ablation experiment, Page 14):**

*In the ablation study, we evaluated the contribution of various components to the overall model performance by removing or replacing key elements. Specifically, we designed two variant models:*

- ***Ours-BiLSTM****: In this variant, the BiLSTM layer was removed, leaving the multi-head attention mechanism. The experiment aimed to assess the attention mechanism’s role in capturing key information from user reviews and course data, focusing on feature interaction and aggregation.*
- ***Ours-Attention****: In this variant, the multi-head attention mechanism was removed, retaining only the BiLSTM layer. The experiment evaluated the BiLSTM layer’s ability to capture sequence dependencies and contextual information, particularly in semantic understanding and feature representation.*

*Based on the experimental results presented in Table 5 and Fig 7, it can be observed that after the removal of both the BiLSTM layer and the attention mechanism, the RMSE and MAE values on both datasets increased, indicating that both components play a significant role in improving the model's performance. However, a comparative analysis reveals that the removal of the BiLSTM layer led to a more substantial decline in performance, particularly with a greater increase in RMSE and MAE on the original dataset. This suggests that the BiLSTM layer contributes more to the model's ability to capture sequence information and semantic dependencies. Therefore, while the attention mechanism is critical for enhancing feature interaction and recommendation accuracy, the BiLSTM layer plays a more crucial role in processing sequence information and semantic understanding.*

**(B，Model architecture, Page 9):**

*Rating prediction is one of the core problems in personalized recommendation systems, aiming to score the interactions between users and courses. The ICRA model predicts user ratings for unseen courses, providing a basis for personalized ranking in the recommendation system. The model predicts scores for all candidate courses and ranks them based on the predicted scores, prioritizing those with higher ratings to achieve personalized recommendations. The ICRA model leverages data collected from the Chinese University MOOC platform, combined with a self-built sentiment lexicon and the pre-trained ERNIE 3.0 model, to conduct in-depth semantic analysis. This model not only accurately identifies and filters fake reviews, ensuring high-quality datasets, but also predicts user ratings for new courses based on their historical rating records. To achieve this, the ICRA model consists of an embedding layer, a feature processing layer, a regularization layer, and an output layer, all of which work closely together to improve rating prediction accuracy.*

*Figure 5 illustrates the overall architecture of the ICRA model, where* $C_{u}$ *represents the user comment document,* $C_{i}$ *represents the course overview document,* $E_{u}$ *and* $E_{i}$ *are the embedding matrices for user and course comment documents, respectively, and* $y_{i}$ *and* $\hat{y_{i}}$ *denote the actual and predicted ratings of users for courses.*

**Major comments 1:**

Figure 1 is seems like a collection of symbols without any textbox describing anything inside the figure or inside the actual text. Authors shoud remove Figure 1 or add appropriate textboxes in the figure and a description in figure caption to make it useful for the reader.

**Response 1:**

Thank you for your valuable comments on our paper. We realize that Figure 1 lacks sufficient descriptive text, which may lead to difficulties for readers in understanding its meaning. To address this issue, we have improved Figure 1 by adding necessary textual explanations, making the content clearer and more comprehensible. Additionally, we have made corresponding adjustments in the main text to ensure coherence and assist readers in better understanding the information conveyed by the figure.To more accurately reflect the content of Figure 1, we have revised the title to "Course Selection Decision Flowchart." This figure illustrates the key factors influencing learners' course selection, and the feedback provided by users after completing a course also helps other users make more informed choices.

***Revised 1 (Introduction, Page 2/10):***

*Learners consider several key factors when selecting a course, such as course content quality, instructor credentials, and user feedback [2]. As shown in Fig 1, after completing the course, users provide feedback, which helps other users make more informed choices.*

**Major comments 2:**

• Figure 3 , please list clearly what is the input to the model and what does the output tensor represent.

**Response 2:**

Thank you for your valuable comments on our paper. In Figure 3, we have clearly outlined the model's inputs and outputs. Specifically, we have added a detailed description of the input tensors to help readers better understand the model's workflow. Additionally, we have modified the related textual explanations to ensure that the overall expression is clearer and more coherent.

**Revised 2 (Related work, Page 4/90):**

*From early statistical models to advanced neural networks like BERT and GPT, pre-trained language models have greatly improved data efficiency, generalization, and enabled transfer learning. While challenges remain in common-sense and logical reasoning, Baidu’s ERNIE series (Enhanced Representation through Knowledge Integration) advances language understanding by integrating knowledge graphs and large-scale corpora. ERNIE 1.0 outperforms BERT in Chinese tasks[21], and ERNIE 2.0 excels in both English and Chinese tasks through multi-task learning[22]. ERNIE 3.0, which integrates knowledge graphs, achieved outstanding results in the 2021 SuperGLUE benchmark[23].*

*As a pre-trained model, ERNIE 3.0 processes tokenized and segmented text sequences by combining word embeddings, position embeddings, and segment embeddings to generate context-aware semantic representations. First, the preprocessed text is fed into the Embedding layer to generate the word embedding vectors W={*$w_{1}$*,* $w_{2}$*,…,* $w_{n}$ *}. Then, the encoding layer extracts features, resulting in an output vector matrix X={*$x_{1}$*,* $x_{2}$*,…,* $x_{n}$*}. This matrix can be used as input for downstream tasks such as text generation, named entity recognition, and text classification. The architecture of the ERNIE 3.0 model is illustrated in Fig 3.*

**Major comments 3:**

• Introduction (page 3/18), the line “Some studies have shown innovative approaches and results” is repeated.

**Response 3:**

Thank you for your valuable feedback. We have removed the repetitive phrase "Some studies have shown innovative approaches and results" from the introduction (page 3/18) and simplified the related statements to ensure that the content of the introduction flows more smoothly and remains consistent.

**Revised 3 (Introduction, Page 3/18):**

*On the other hand, the course review perspective assesses the quality and effectiveness of the course, analyzing factors like review content and course ratings to provide a more precise course profile. ~~Some studies have shown innovative approaches and results.~~ Some studies have demonstrated innovative methods and achievements. For instance, researchers have introduced counterfactual sample enhancement techniques to improve the performance of these systems [18]. Additionally, BERT and Bi-GRU networks have been combined to learn semantic features and sentiment weights from review texts [19]. Moreover, a novel Review-based Graph Comparison Learning (RGCL) framework has been proposed to further enhance recommendation accuracy [20].*
